# The Mechanism of Wear Reduction in the Ni-CaF_2_ Composite Material: Raman and Confocal Microscopy Insights

**DOI:** 10.3390/ma15165501

**Published:** 2022-08-10

**Authors:** Mateusz Kotkowiak, Adam Piasecki, Michał Kotkowiak, Tomasz Buchwald

**Affiliations:** 1 Institute of Materials Engineering, Faculty of Materials Engineering and Technical Physics, Poznan University of Technology, Jana Pawła II 24, 60-965 Poznan, Poland; 2 Institute of Physics, Faculty of Materials Engineering and Technical Physics, Poznan University of Technology, Piotrowo 3, 60-965 Poznan, Poland; 3 Institute of Materials Research and Quantum Engineering, Faculty of Materials Engineering and Technical Physics, Poznan University of Technology, Piotrowo 3, 60-965 Poznan, Poland

**Keywords:** calcium fluoride, self-lubricating material, surface analysis, Raman spectroscopy, confocal microscopy

## Abstract

A powder metallurgy process was used to produce high temperature self-lubricating composites based on Ni, with varying content of calcium fluoride (10 wt.% and 20 wt.%). The wear properties of the samples were investigated by a pin-on-disc test at elevated temperature, up to 600 °C. Aside from standard techniques for the sample characterization, confocal microscopy and micro-Raman spectroscopy were used for the first time for this type of sample. These methods were used to examine the changes in topography and to detect the distribution of the tribofilm on sample surfaces. The addition of solid lubricant particles decreased the coefficient of friction and improved the tribological properties, because of the tribofilm which formed on sample surfaces.

## 1. Introduction

Self-lubricating materials are very popular nowadays because of their good tribological properties, such as high wear resistance and low friction coefficient. There are many methods which are used for producing this type of material, for example: thermal explosion [1], magnetron sputtering [2,3], powder metallurgy [4,5,6,7], laser alloying [8,9] and laser cladding [10,11,12,13,14,15,16,17]. Self-lubricating ceramic composites have been widely used in different applications, because these materials combine good properties of the matrix and the solid lubricant. Furthermore, in ceramic matrix composites, solid lubricants are evenly distributed in the matrix [18]. Wear properties of self-lubricating ceramic materials depend on the chemical composition of the matrix and the type of solid lubricant. Both have an influence on tribological properties and can easily change friction conditions. In the design process of self-lubricating composites, the most important thing is to predict the temperature in which the composite will be working. Therefore, the correct self-lubricating materials should be used. Self-lubricating materials are grouped into three groups which differ in operating temperature [19]. The range of work temperature of the first group is: −200 °C to room temperature, second group—room temperature to 500 °C and the third group—above 500 °C. Currently, the high temperature solid-lubricant materials calcium fluoride (CaF_2_) [20,21,22,23,24] and barium fluoride (BaF_2_) [25,26,27] are commonly used [28]. These lubricants are characterized by very important properties, such as: high chemical and thermal stability [29,30], low density and beneficial crystal structure which ensure good lubricating properties [31,32]. Moreover, carbon-based lubricants such as DLC (diamond-like carbon coatings), nanotubes (CNT), or graphene are very important [33,34,35]. Aside from metal-based self-lubricating materials, solid lubricants are commonly used in metal-free polymer composites. Good examples of such materials are polyimide composites doped with graphene and nano-MoS_2_ [36] or nano-graphite [37,38]. It is also very popular to produce self-lubricating polymer coatings where, as a lubricant, another polymer is used [39]. Therefore, invention of new materials, which would be characterized by high wear resistance, is desirable. Aside from the manufacture of modern self-lubricating materials, excellent knowledge of wear mechanisms is also important if researchers want to reduce frictional losses. Study of the wear mechanism is possible because modern and interdisciplinary techniques, such as confocal microscopy and Raman spectroscopy, can be used [40,41,42]. In this study, Ni composites with addition of CaF_2_ were produced. Powder metallurgy, which combined the pressing and sintering, was used to produce the self-lubricating materials. Sintering was carried out in an inert atmosphere (Ar) at temperature equal to 1200 °C for 2 h. The wear properties were investigated in the range of room temperature to 600 °C. To gain deeper insight into the wear mechanism, modern and interdisciplinary techniques, such as confocal microscopy and Raman spectroscopy, were used. The use of these techniques provided a better understanding of the wear process and the lubrication process. 

## 2. Experimental Methods

### 2.1. Powder Metallurgy

Self-lubricating composites were produced by powder metallurgy. The nickel powder was characterized by the spherical form of the particles and the dimensions were lower than 5 µm. The CaF_2_ was characterized by a cube-like shape. The size of the particles was less than 10 µm. The first step was to prepare the powder mixtures: Ni, Ni with 10 wt.% CaF_2_ and Ni with 20 wt.% CaF_2_. The images of the powders used in this paper are shown in Supplementary Materials (Figure S1). Powder mixtures were pressed, and the pressure was 11,942 kgf/cm^2^ (1.17 GPa). Sintering was conducted in the furnace with an inert (argon) atmosphere. The sintering was conducted for 2 h, and the sintering temperature was 1200 °C.

### 2.2. Wear Tests

Wear tests were performed on the pin-on-disc tribometer (T21, Lukasiewicz Research Network—IST, Radom, Poland) which was equipped with control module, displacement and friction force sensors. The control module was used to set the temperature and the rotational speed of the counter-specimen, whereas the sensors recorded the specimen displacement and friction force. The sample was placed in a special holder. The samples were 4 mm in diameter and 6 mm in height. The Inconel^®^ 625 (purchased from Bibus Metals, Dąbrowa, Poland) alloy was used as the counter-specimen, which was characterized by the diameter of 25 mm and height of 10 mm. The counter-specimen’s chemical composition is shown in Table 1. The counter-specimen was mounted on the rotational platform. The parameters of the tribotest, which was conducted under dry friction conditions, were as follows: rotational speed of the counter-specimen—120 min^−1^, load—F = 4.9 N, time—60 min and different temperatures (23 °C, 200 °C, 400 °C and 600 °C). The experiment was repeated three times. After the wear test, the friction coefficient, µ, was calculated from the following equation:

$$\mu =\frac{F_{f}}{F_{n}},$$

where: *μ*—friction coefficient, *F_f_*—friction force [N]; *F_n_*—applied load [N]. 

The wear rate was calculated as the total linear wear of the sample and counter-sample per hour.

### 2.3. SEM and EDS Analysis

A scanning electron microscope (Tescan Vega 5135, Brno, The Czech Republic) was used in order to investigate the surface conditions of the samples after the wear tests. The EDS method was used to analyze the worn surfaces of the samples. A microanalyzer (PGT Avalon), with 55° take-off angle, was used. This method allows to study the distribution of sample characteristic elements. Nickel, chromium, calcium and fluorine were investigated. An accelerating voltage of 12 kV was used. The previous study showed that the tribofilm layer was very thin, therefore such a low voltage was used. Higher voltage might give results from the higher excitation zone, thus making detection of calcium and fluorine more difficult.

### 2.4. Confocal Microscopy

A laser confocal microscope (Carl Zeiss LSM 710, Jena, Germany) was used in order to reveal the stereometric profile of the counter-specimen. The microscope was equipped with a HeNe laser with a wavelength equal to 543 nm. On one side of the figures the base material was shown and on the other side the frictional path could be clearly seen. The stereometric profiles give information about the differences in the heights of the samples.

### 2.5. Raman Spectroscopy

The Raman spectroscopy analysis was carried out to detect the tribofilm distribution on the surface of the samples after the wear test in different temperatures. The inVia Raman microscope (Renishaw, Wotton-under-Edge, UK) with diode-pumped laser with a wavelength of 785 nm was used. The samples with the addition of the solid-lubricant were investigated. The size of the Raman maps was 50 µm × 50 µm and the measurements were carried out every 2 µm in each direction.

## 3. Results and Discussion

### 3.1. Wear Tests

The detailed analysis of the wear test for pure Ni and self-lubricating composite with addition of 20% of CaF_2_ was described in a previous publication [43]. In this section the results of the wear test for the sample with addition of 10% CaF_2_ are presented. 

The detailed results of the wear test performed at 23 °C are shown in the Supplementary Materials (Figure S2). The first stage, grinding-in, lasted approximately 1100 s, in comparison with 750 s for pure Ni and 790 s for Ni-20% CaF_2_. Then, the average friction coefficient, *µ_a_*, was equal to 0.72. However, after 3250 s the friction coefficient increased and after that time *µ_a_* was equal to 0.74. Furthermore, some large fluctuations of the friction coefficient were observed after 1400 s, which was probably caused by a break in the tribofilm layer produced on the surface of the sample. The observations of the friction coefficient variability indicated that the Ni-20% CaF_2_ self-lubricating composite was better despite a slightly higher value of average friction coefficient (0.76).

In the Supplementary Materials (Figure S3) the detailed results of the wear test conducted at 200 °C are shown. The grinding-in lasted approximately 945 s and after that, the *µ_a_* value was equal to 0.76. In this temperature the *µ_a_* for Ni-10% CaF_2_ was lower than *µ_a_* for pure Ni (0.87) and higher than *µ_a_* for Ni-20% CaF_2_ (0.72). Moreover, some fluctuations of the course of the µ were visible. It could indicate that the tribofilm layer was not continuous, as in the case of the Ni-20% CaF_2_ self-lubricating composite.

In the case of the wear test conducted at 400 °C (Supplementary Materials, Figure S4), the grinding-in lasted approximately 900 s, which was longer than for the self-lubricating composite with the addition of 20% calcium fluoride (500 s). After that time the *µ* was stable over time and the *µ_a_* was equal to 0.72. Furthermore, temporary increases of *µ* were observed e.g., 2250 s, 2560 s, 3000 s and 3400 s. These temporary increases were probably related to breaking the tribofilm layer.

In the Supplementary Materials (Figure S5) the results of the wear test conducted at 600 °C are shown. The first stage of the wear process, for the sample containing 10% CaF_2_, lasted approximately 850 s. After that time the *µ_a_* was equal to 0.68. After 1900 s the *µ* decreased significantly and the *µ_a_* was equal to 0.47. A decrease in the average friction coefficient after 1900 s could be explained by a long production time of the thin tribofilm layer. As found in the previous work [43], the µ_a_ for pure Ni and self-lubricating composite Ni-20% CaF_2_ was 0.69 and 0.46, respectively.

**Figure 1:**
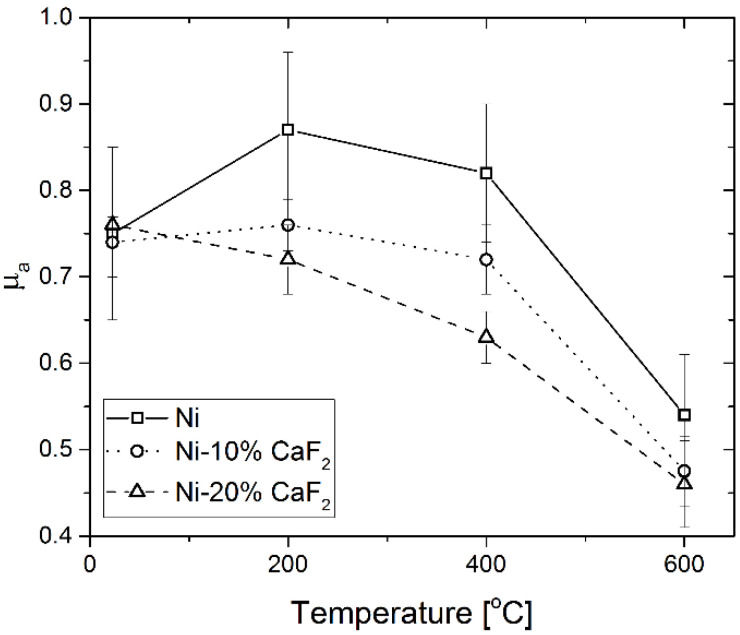
The average values of the friction coefficient (*µ_a_*) vs. temperature of friction for pure Ni, sintered Ni-10% CaF_2_ and sintered Ni-20% CaF_2_ self-lubricating composite mating with Inconel^®^625-alloy. Results for pure Ni and for 20% addition of CaF_2_ were taken from previous work [43] and added for data clarity.

**Figure 2:**
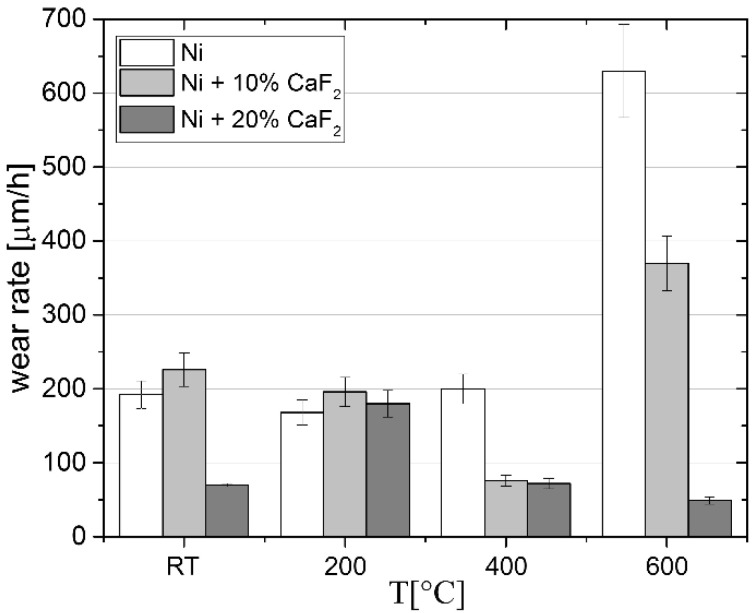
Wear rates at investigated temperature for sintered pure Ni, Ni-10% CaF_2_ and Ni-20% CaF_2_ self-lubricating composites. Results for pure Ni and for 20% addition of CaF_2_ were taken from previous work [43] and added for data clarity.

The *µ_a_* values of the investigated materials are presented in Figure 1. The values were comparable only at room temperature, probably because of the calcium fluoride, which is a high-temperature solid lubricant and has good lubricating properties at elevated temperatures. Therefore, after the wear tests carried out at 200 °C, 400 °C and 600 °C, the differences in the *µ_a_* for pure Ni and self-lubricating composites were significant. Moreover, the higher values of the average friction coefficient were observed for the Ni-10% CaF_2_ than for the Ni-20% CaF_2_ at 200 °C and 400 °C, but at 600 °C the values of *µ_a_* were similar (0.46 vs. 0.47). The composites with the addition of CaF_2_ were characterized by lower values of the friction coefficient because of the beneficial crystal structure of the lubricant. The lamellar structure causes a lower shear strength which also decreases with temperature, and calcium fluoride smeared on the surface of the sample with ease [44,45,46]. Furthermore, the wear rates of the frictional pair (entire linear wear of the frictional pair) are presented in Figure 2. The wear rate for pure Ni increased with the temperature and reached maximum at 600 °C. The situation changed for the self-lubricating composites. At room temperature the wear rates for pure Ni and Ni-10% CaF_2_ were comparable, whereas the wear rate for Ni-20% CaF_2_ was approximately equal to 70 µm/h. At 200 °C, the wear rates for all materials were similar. The lowest wear rate was obtained for the self-lubricating composite containing 20% CaF_2_ at 600 °C.

**Figure 3:**
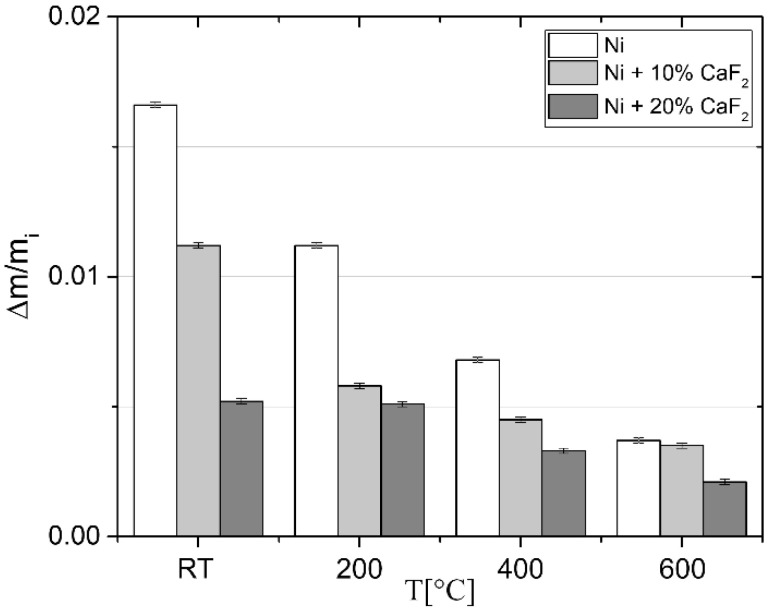
Relative loss of mass of the counter-samples.

Moreover, the wear rate of the counter-samples is very important. The relative losses of mass of the counter samples are shown in Figure 3. The wear rate was calculated from the following equation:

(1) $$\frac{∆m}{m_{i}}=\frac{m_{i}-m_{f}}{m_{i}},$$

where:

- ∆*m*—mass loss (mg),
- *m_i_*—initial mass of counter-sample (mg),
- *m_f_*—final mass of counter-sample (mg).

The relative loss of mass of the counter-samples decreased at elevated temperature (Figure 3). Moreover, the relative loss of mass of counter-samples increased when the counter-sample mated with pure Ni. On the other hand, the relative loss of mass of the counter-sample mated with self-lubricating composites diminished when the CaF_2_ amount increased. It means that the tribofilm which was produced between the self-lubricating composites and the counter-sample protected the frictional pair against wear.

### 3.2. Worn Surface Analysis

**Figure 4:**
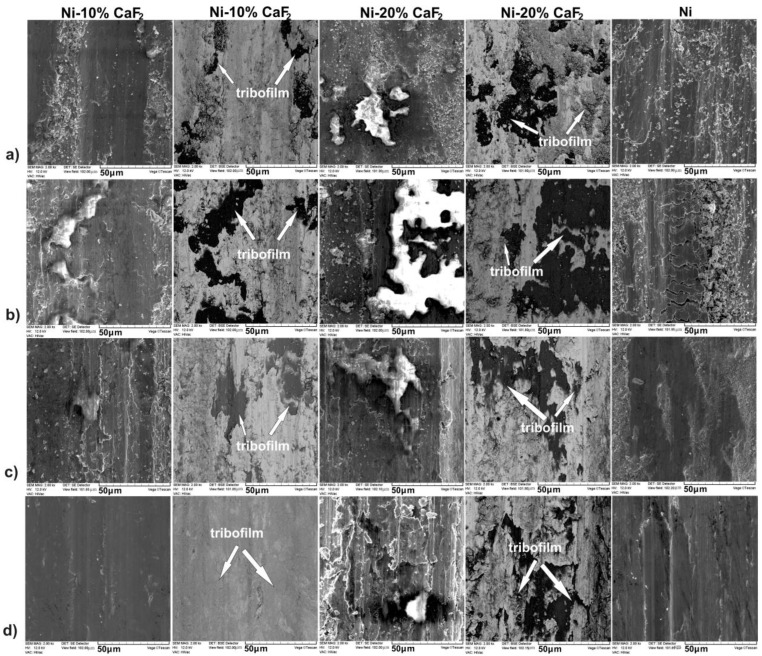
The surface of the worn samples tested at 23 °C (**a**), at 200 °C (473 K) (**b**), at 400 °C (673 K) (**c**), at 600 °C (873 K) (**d**). Results for pure Ni and for 20% addition of CaF_2_ were taken from previous work [43] and added for data clarity. White arrows indicate places where the tribofilm was produced during friction.

Surface analyses of the sintered materials, after wear, were performed by the SEM, EDS method, laser confocal microscope and Raman spectroscopy. The detailed description of the surfaces of the pure Ni and Ni-20% CaF_2_ self-lubricating composite can be found in the work [43]. In Figure 4 the worn surfaces of the samples are presented. The surface of the Ni-10% CaF_2_ self-lubricating composites was coated by the tribofilm, which was characterized by irregular thickness. The irregular thickness of the tribofilm was clearly visible on the BSE pictures, due to the differences in the grayscale. Furthermore, the calcium fluoride particles could be easily noticed on the surface of the samples. In the case of the pure Ni, intensive abrasive wear occurred, which was manifested by shallow grooves and cracks.

**Figure 5:**
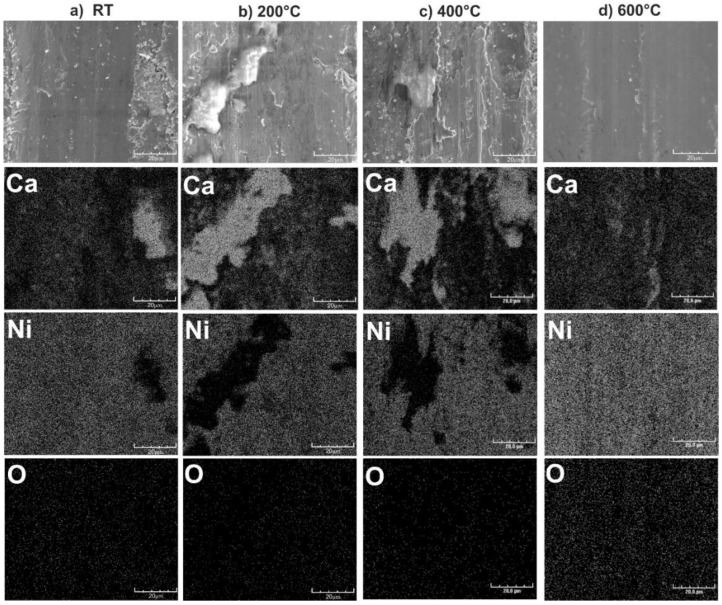
The EDS analysis of the surface of the samples after wear tests of Ni-10% CaF_2_ self-lubricating composites at different temperatures.

The EDS patterns from the worn surfaces of the Ni-10% CaF_2_ self-lubricating composite are shown in Figure 5. The distribution of calcium, nickel and oxygen was analyzed. The EDS analysis proved formation of the tribofilm on the surfaces of the samples, which could be seen on the calcium concentration images. In the places where calcium was present a lower amount of nickel was detected. The worn surface analysis of all the samples also showed that more calcium was on the surfaces of the samples with 20% addition of CaF_2_ and, because of that, lower values of the friction coefficient and wear rate were obtained during wear tests. The increased content of oxygen was observed only for the sample tested at 600 °C, which was probably related to the oxidation of the nickel.

**Figure 6:**
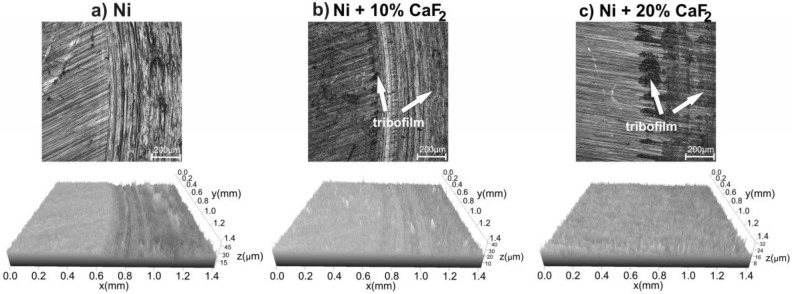
Stereometrics profiles of counter-specimens mated with produced sinters, after wear test at 23 °C.

**Figure 7:**
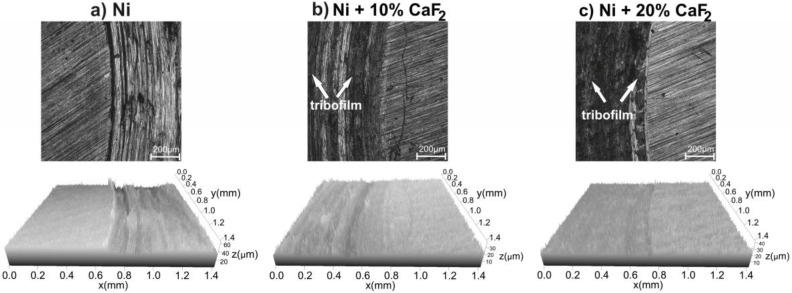
Stereometrics profiles of counter-specimens mated with produced sinters, after wear test at 200 °C.

**Figure 8:**
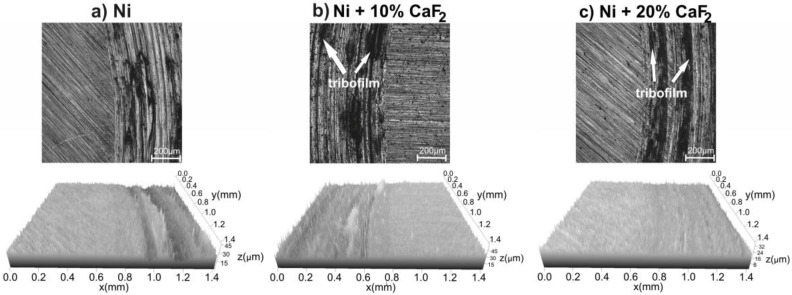
Stereometrics profiles of counter-specimens mated with produced sinters, after wear test at 400 °C.

**Figure 9:**
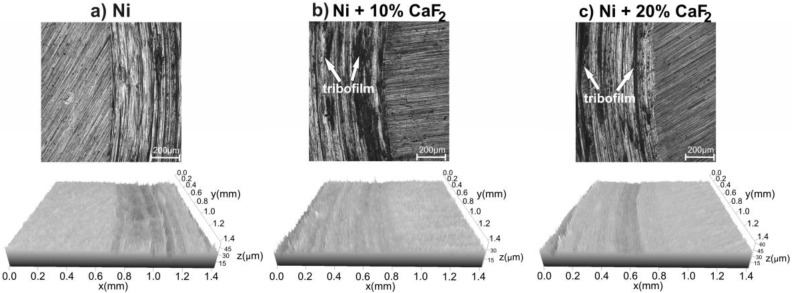
Stereometrics profiles of counter-specimens mated with produced sinters, after wear test at 600 °C.

Stereometric profiles of the surfaces of the counter-samples are presented in Figure 6, Figure 7, Figure 8 and Figure 9. In the figures, the base material and the frictional path are shown. Because of that, the differences in the stereometric profiles of the counter-specimens can be clearly seen. After the wear test, which was carried out at room temperature, the surfaces of the counter-specimen were different from each other (Figure 6). The surface of the counter-specimen which was mated with pure Ni was very rough, and some deep grooves were visible (Figure 6a). On the other end was the counter-specimen which made a frictional pair with the self-lubricating composites containing 20% calcium fluoride (Figure 6c). The surface of the counter-specimen was smooth and the height differences between base material and the frictional path were very small. Stereometrics profiles for the counter-samples worn at 200 °C are shown in Figure 7. On the profile characteristic for the counter-specimen mated with pure Ni, a lot of deep grooves could be noticed and also the frictional path was very rough. In the case of the second counter-sample (Figure 7b) the grooves were visible, but they were not as deep as in the first case. The smoothest surface of the counter-specimen was obtained in the last case, when the counter-specimen worked with the Ni-20% CaF_2_ self-lubricating composite (Figure 7c). When the temperature of the wear study was equal to 400 °C the stereometric profiles of the counter-specimen (Figure 8) were very similar to the previous, which are shown in Figure 7. The smoothest profile was characteristic for the counter-specimen mated with Ni-20% CaF_2_ self-lubricating composite (Figure 8c). Additionally, the signs of the adhesive wear were visible in Figure 8b. In Figure 9 the stereometric profiles obtained at the 600 °C are shown. The first profile (Figure 9a) was very rough, and deep grooves were visible. The stereometrics profiles of the counter-specimen mated with Ni-10% CaF2 and Ni-20% CaF_2_ self-lubricating composites were very similar. They were smooth and some shallow grooves were visible. In the pictures of the counter-specimen’s surface, mated with the self-lubricating composites, the signs of the tribofilm were visible (Figure 6b,c, Figure 7b,c, Figure 8b,c and Figure 9b,c). It can be easily noticed that the tribofilm was smeared on the surface of the counter-specimen.

**Figure 10:**
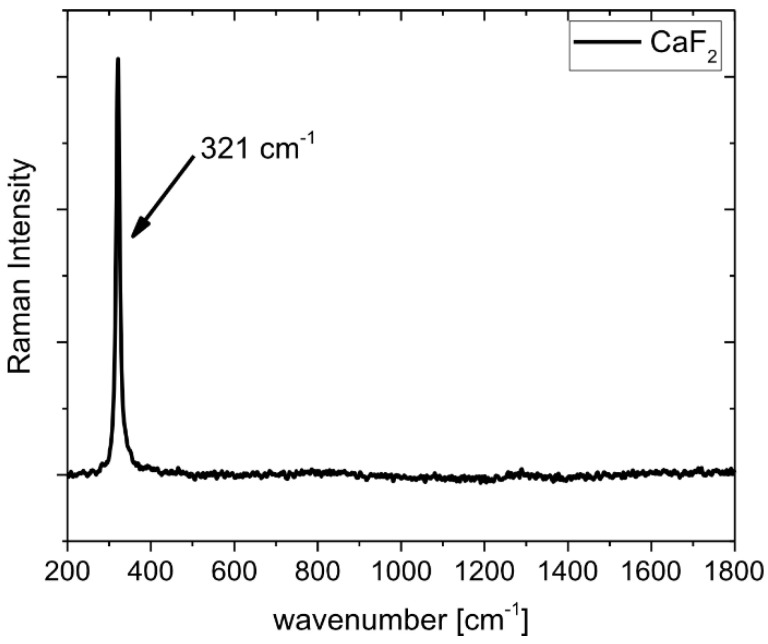
Raman spectrum of CaF_2_.

**Figure 11:**
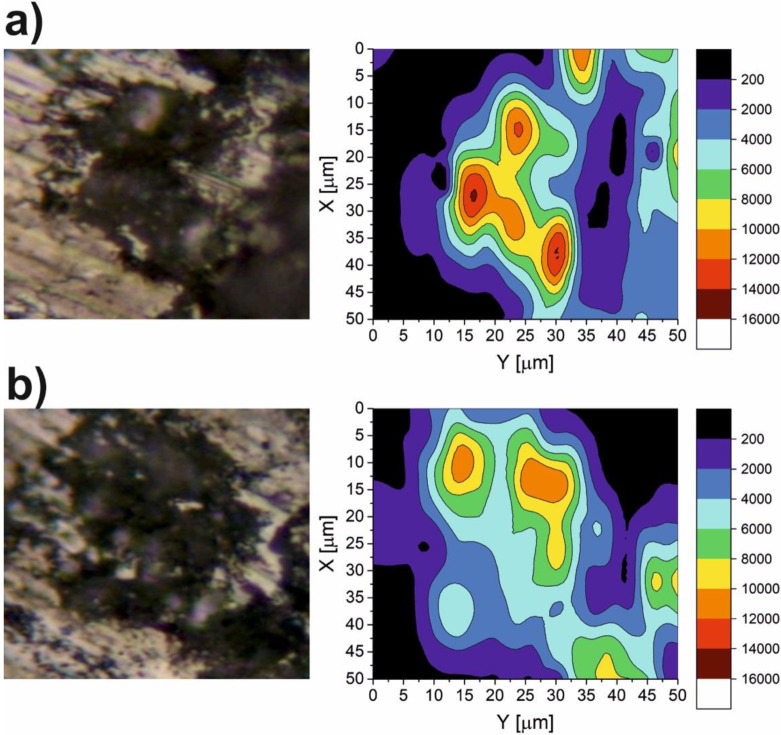
The Raman maps of Ni-10% CaF_2_ (**a**) and Ni-20% CaF_2_ (**b**) self-lubricating composites after wear test at room temperature.

**Figure 12:**
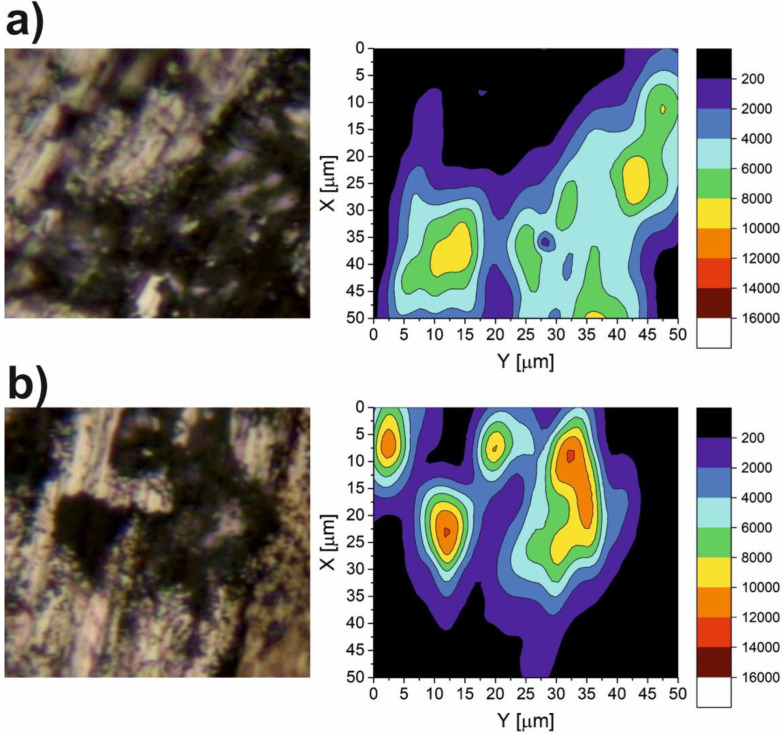
The Raman maps of Ni-10% CaF_2_ (**a**) and Ni-20% CaF_2_ (**b**) self-lubricating composites after wear test at 200 °C.

**Figure 13:**
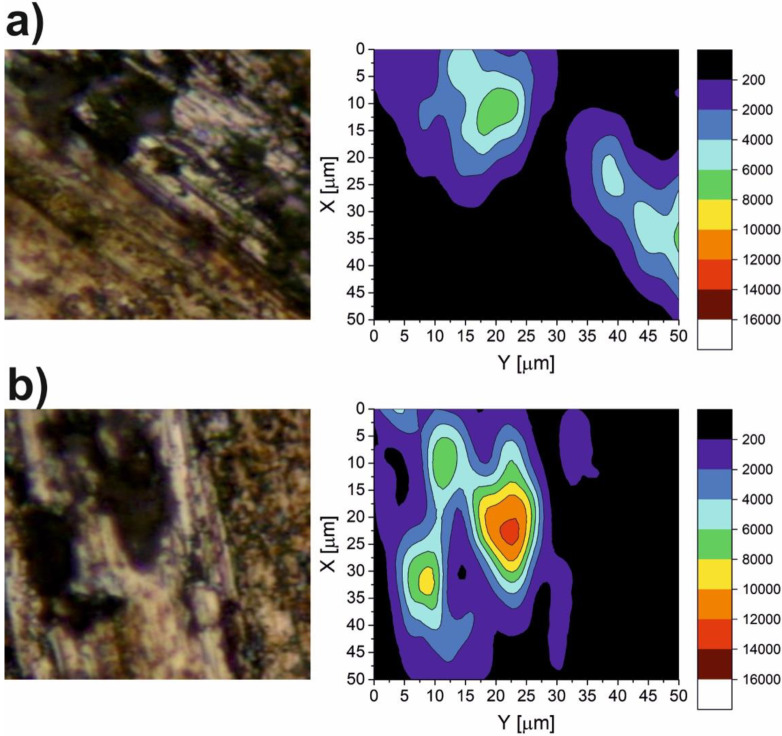
The Raman maps of Ni-10% CaF_2_ (**a**) and Ni-20% CaF_2_ (**b**) self-lubricating composites after wear test at 400 °C.

**Figure 14:**
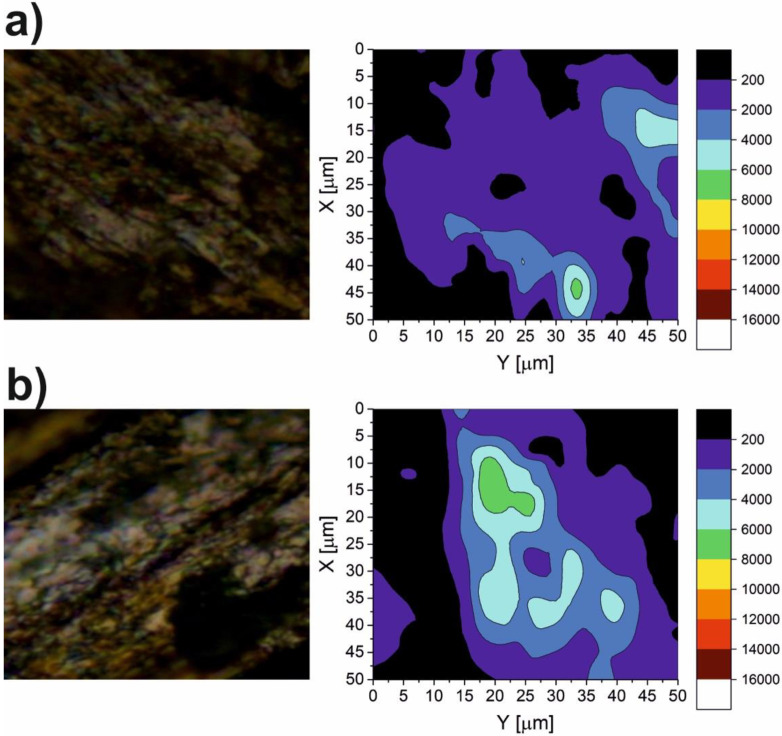
The Raman maps of Ni-10% CaF_2_ (**a**) and Ni-20% CaF_2_ (**b**) self-lubricating composites after wear test at 600 °C.

The results of the micro-Raman spectroscopy are shown in Figure 10, Figure 11, Figure 12, Figure 13 and Figure 14. The Raman spectra for the CaF_2_ powder are presented in Figure 10. The spectrum for the CaF_2_ was characterized by the appearance of a peak at 321 cm^−1^. Therefore, the micro-Raman maps (Figure 11, Figure 12, Figure 13 and Figure 14) showed intensity changes at wavenumber equal to 321 cm^−^^1^. In the case of the wear test conducted at room temperature, maps for the sample containing 10% CaF_2_, showed more areas with higher intensity than the maps characteristic for the sample with 20% CaF_2_. The situation changed at 200 °C and 400 °C. In these cases, higher intensity areas appeared on the Ni-20% CaF_2_ sample, which meant that the tribofilm was characterized by a higher thickness. Additionally, the values of the *µ_a_* were lower for the nickel modified by 20% CaF_2_. The maps obtained for the samples worn at 600 °C were similar to each other. The sizes of the tribofilm areas and the intensity were comparable. Because of that, the values of the *µ_a_* were almost equal: 0.47 vs. 0.46. The results of the Raman spectroscopy showed, that the tribofilm was made of CaF_2_. This research method proved that the CaF_2_ has high chemical and thermal stability, because it occurred on the surface of the sample as the same chemical compound which was used to produce the samples. The Raman spectroscopy is a very useful method, compared to, e.g., EDS method, because it can detect the chemical compounds which are formed on the surface of the sample during wear, whereas the EDS method, which is widely used to analyze the wear of the materials, detects only the elements that appear on the surface of the sample.

**Figure 15:**
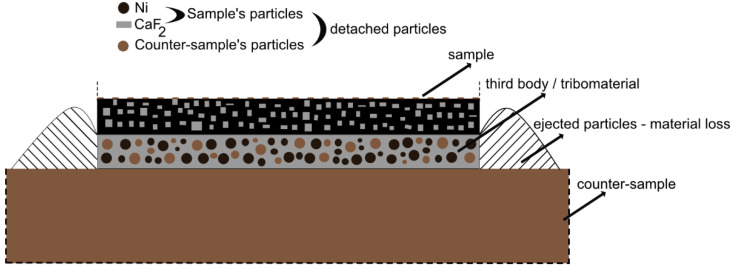
The third body concept in the investigated pure Ni/self-lubricating composites system.

In this system, the third body concept developed by N. Fillot could be applied [47], for a better understanding and complete description of the wear process. Moreover, that third body model was studied by other authors in order to explain the fretting fatigue in the 35 NiCrMo16/52 100 steel interface [48,49] or tribological behavior of an AISI 316L stainless steel [35,50]. During wear process, a large number of detached particles was generated from the surface of the sample and counter-sample. Moreover, some of those particles were ejected from the system (frictional pair) and formed the wear debris which was the wear loss. However, according to the third body concept, the total mass of detached and ejected particles is not the same, which means that between the frictional pair a certain amount of mass remains that is called third body. In a pure Ni/self-lubricating composites frictional pair, the third body contained a high amount of solid lubricant (CaF_2_) besides other particles, which was confirmed by EDS, Raman spectroscopy and LCM methods. The scheme of this concept is shown in Figure 15. Furthermore, the tribofilm was responsible for lower values of friction coefficient, wear rates and also lower wear loss. It was impossible to remove the entire third body from the system (sample/counter-sample) because the tribofilm was characterized by excellent adhesion to both materials, which was clearly seen on the LCM figures of the counter-samples (Figure 6, Figure 7, Figure 8 and Figure 9). Because of that, the wear rate (Figure 2) was calculated previously as an entire linear wear of the frictional pair. Moreover, the thin tribofilm layer was renewed constantly because the ejected particles were instantly replaced by the detached ones.

## 4. Conclusions

**Figure 16:**
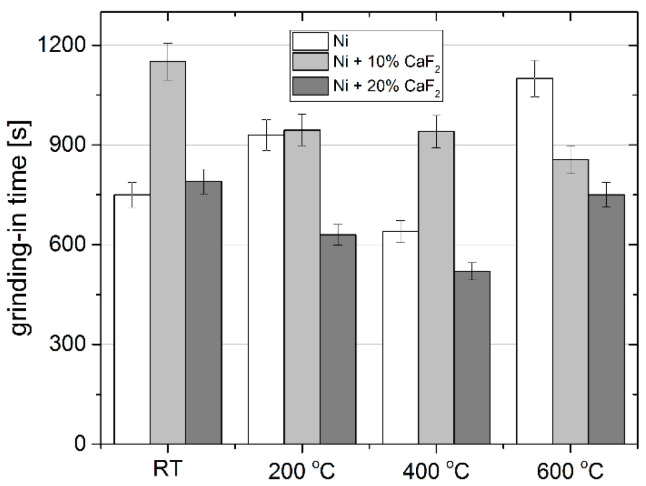
The grinding-in times for sintered Ni, Ni-10% CaF_2_ and Ni-20% CaF_2_ self-lubricating composites after wear test at different temperatures.

In this paper self-lubricating composites, Ni-10% CaF_2_ and Ni-20% CaF_2_, were studied. Wear tests conducted at different temperatures confirmed beneficial lubrication properties of calcium fluoride. During the test tribofilm was produced on the surface of the sample and caused a decrease in the friction coefficient. At 23 °C the µa for every sample was similar and the lowest value was obtained for the Ni modified by the addition of 10% CaF_2_, whereas, at elevated temperature the Ni-20% CaF_2_ self-lubricating composite was characterized by the lowest value of *µ_a_*. Furthermore, the lowest value of the wear rate was obtained for the sample containing 20% of CaF_2_ at every investigated temperature. Moreover, the duration of the first stage of the wear—grinding-in—was diminished, by addition of 20% CaF_2_, even to 25% compared to other samples (Figure 16). EDS analysis showed a higher concentration of calcium in areas where the tribofilm was produced. It meant, that during wear, the tribofilm was smeared on the surface of the samples containing CaF_2_. The stereometrics profiles showed that the surfaces of the counter-specimens mated with the self-lubricating composites were smooth and it indicated that the tribofilm protected the sample as well as the counter-sample from wear.

The presence of the tribofilm ensured that sample surfaces were smoother without any deep scratches, which were observed on the surfaces of the pure Ni after wear test conducted at room and at elevated temperatures. The intensive abrasive wear was identified as a main wear mechanism for the pure nickel samples. On the other side, where the self-lubricating composites were coated by the tribofilm, the intensive abrasive wear did not occur. EDS analysis and Raman spectroscopy showed that the tribofilm was characterized by diversified thickness, which had an influence on the tribological properties. With the use of Raman mapping, we were able to correlate the observed Raman intensity of CaF_2_ with the process temperature and, more importantly, with the friction coefficients. The Raman spectroscopy showed that the maps intensity for the self-lubricating composites was comparable at room temperature and at 600 °C. Because of that, at these temperatures, the friction coefficients were similar. At 200 °C and 400 °C the situation changed: the Raman maps obtained higher intensity for Ni-20% CaF_2_ self-lubricating composite, which caused the lower values of the *µ*. Finally, we proposed the third body concept in the investigated pure Ni/self-lubricating composite system. Due to the superb adhesion of the tribofilm to both materials, it was impossible to remove the entire third body from the system. Moreover, the tribofilm is constantly renewed because of the replacement of ejected particles with detached ones.

## Figures and Tables

**Table 1:** Chemical composition of counter-specimen material (wt.%).

Material	Ni	Cr	Mo	Nb + Ta	Fe	C	Mn	Si	S	Al	Ti	P	Co
Inconel ^®^625	balance	22	8.6	3.6	4.1	0.03	0.06	0.02	0.001	0.2	0.2	0.005	0.13

## Data Availability

The data presented in this study are available on request from the corresponding author.

## Supplementary Materials

The following supporting information can be downloaded at: https://www.mdpi.com/article/10.3390/ma15165501/s1, Figure S1: The powders used in this investigation: pure Ni (a) and CaF_2_ (b). Figure S2: Friction coefficient vs. time of friction for sintered Ni-10%CaF_2_ self-lubricating composite, mating with Inconel^®^625-alloy at room temperature. Figure S1: Friction coefficient vs. time of friction for sintered Ni-10%CaF_2_ self-lubricating composite, mating with Inconel^®^625-alloyat 200 °C (473 K). Figure S4: Friction coefficient vs. time of friction for sintered Ni-10% CaF_2_ self-lubricating composite, mating with Inconel^®^625-alloy at 400 °C (673 K). Figure S5: Friction coefficient vs. time of friction for sintered Ni-10% CaF_2_ self-lubricating composite, mating with Inconel^®^625-alloy at 600 °C (873 K).
