# Supplementary material for: The Mechanism of Wear Reduction in the Ni-CaF2 Composite Material: Raman and Confocal Microscopy Insights
Source: Materials (Basel). 2022 Aug 10;15(16):5501. doi: 10.3390/ma15165501 (PMC9413760; doi:10.3390/ma15165501)
Supplement: Supplementary file 1 [file materials-15-05501-s001.zip › materials-1804218-supplementary.pdf]

Supplementary material
for

The mechanism of wear reduction in the Ni-CaF_2 composite material: Raman and confocal microscopy insights

Mateusz Kotkowiak[a*], Adam Piasecki[a], Michał Kotkowiak[b], Tomasz Buchwald[c]

[a] *Institute of Materials Science and Engineering, Poznan University of Technology, Pl. M. Sklodowskiej-Curie 5, 60-965 Poznan, Poland*
[b] *Institute of Physics, Poznan University of Technology, Piotrowo 3, 60-965 Poznan, Poland*
[c] *Institute of Materials Research and Quantum Engineering, Poznan University of Technology, Piotrowo 3, 60965 Poznan, Poland*

*corresponding author mateusz.kotkowiak@put.poznan.pl (Mateusz Kotkowiak)

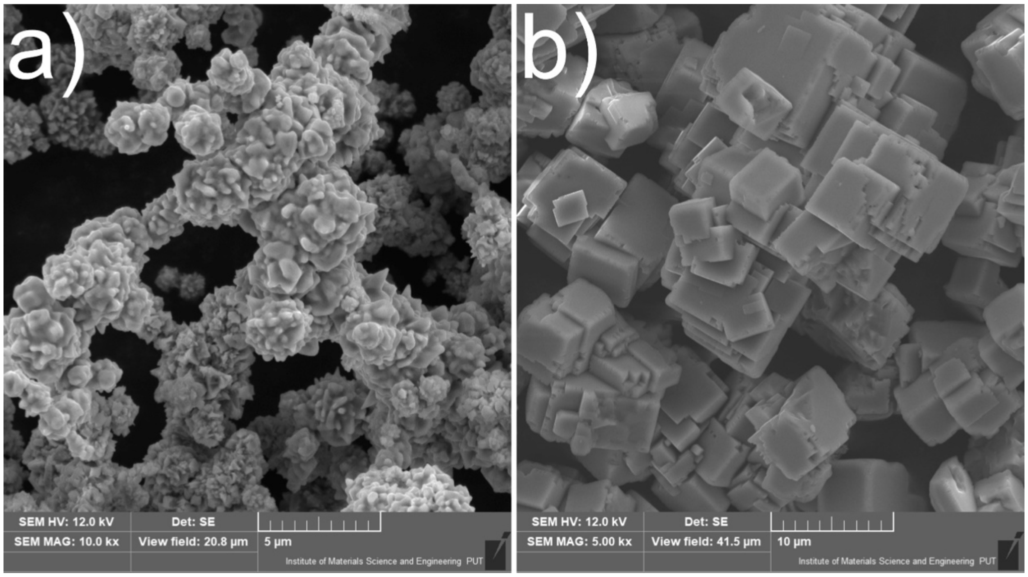

Figure S1. The powders used in this investigation: pure Ni (a) and CaF_2 (b).

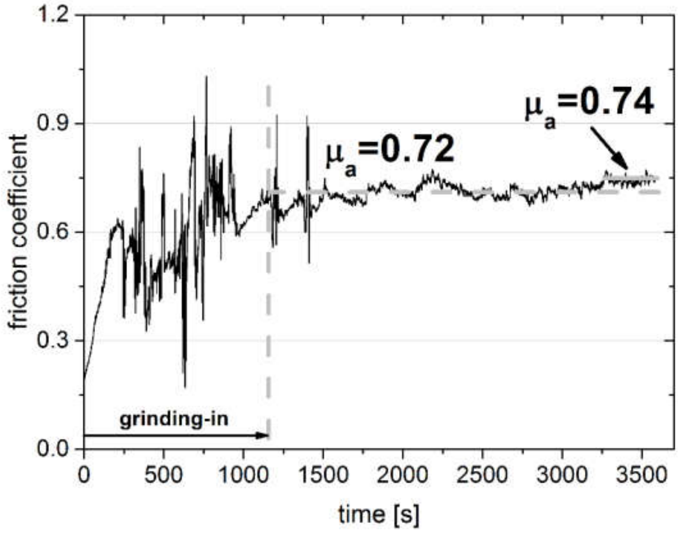

Figure S2. Friction coefficient vs. time of friction for sintered Ni-10%CaF_2 self-lubricating composite, mating with Inconel®625-alloy at room temperature.

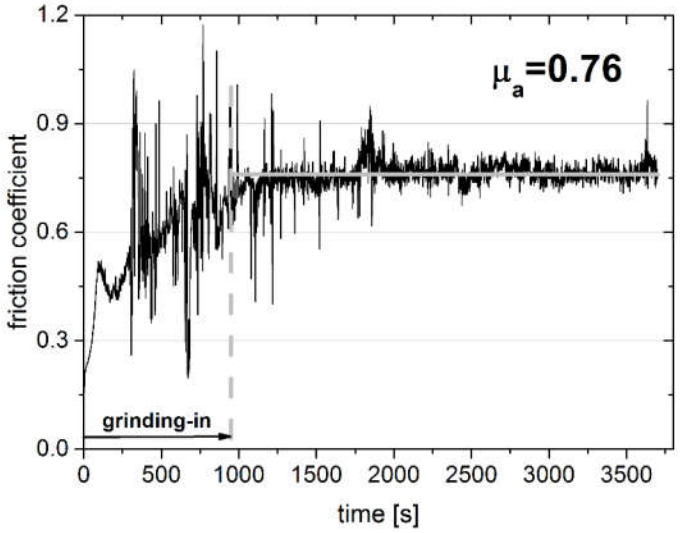

Figure S3. Friction coefficient vs. time of friction for sintered Ni-10%CaF_2 self-lubricating composite, mating with Inconel®625-alloy at 200ºC (473 K).

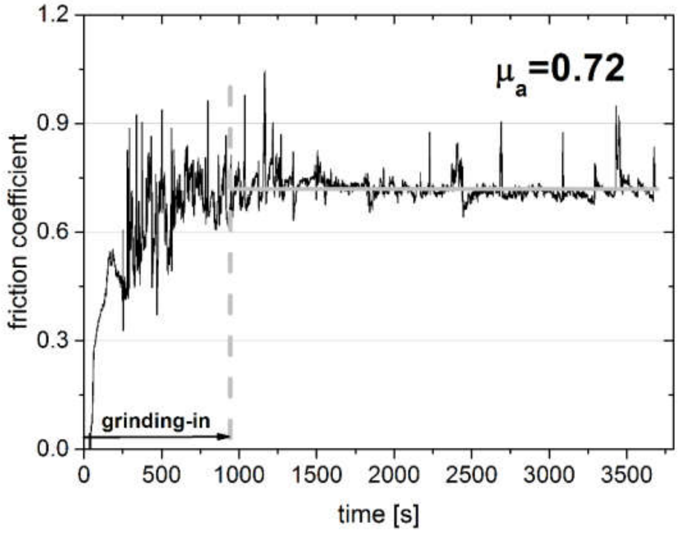

Figure S4. Friction coefficient vs. time of friction for sintered Ni-10%CaF_2 self-lubricating composite, mating with Inconel®625-alloy at 400ºC (673 K).

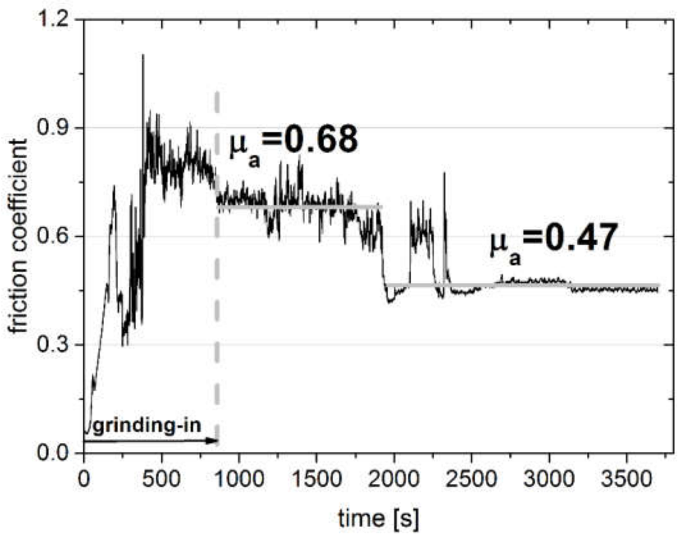

Figure S5. Friction coefficient vs. time of friction for sintered Ni-10%CaF_2 self-lubricating composite, mating with Inconel®625-alloy at 600ºC (873 K).
